# Comparative Susceptibility of Different Biological Forms of *Anopheles stephensi* to *Plasmodium berghei* ANKA Strain

**DOI:** 10.1371/journal.pone.0075413

**Published:** 2013-09-23

**Authors:** Hamid R. Basseri, Habib Mohamadzadeh Hajipirloo, Mulood Mohammadi Bavani, Miranda M. A. Whitten

**Affiliations:** 1 Department of Medical Entomology and Vector Control, School of Public Health, Tehran University of Medical Sciences, Tehran, Iran; 2 Department of Parasitology and Mycology, Faculty of Medicine, Urmia University of Medical Sciences, Urmia, Iran; 3 Institute of Life Science, College of Medicine, Swansea University, Singleton Park, Swansea, United Kingdom; Centro de Pesquisas René Rachou, Brazil

## Abstract

**Background:**

There are varying degrees of compatibility between malaria parasite-mosquito species, and understanding this compatibility may be crucial for developing effective transmission-blocking vaccines. This study investigates the compatibility of different biological forms of a malaria vector, *Anopheles stephensi*, to *Plasmodium berghei* ANKA strain.

**Methods:**

Several biologically different and allopatric forms of *A. stephensi* were studied. Three forms were isolated from different regions of southern Iran: the variety mysorensis, the intermediate form and the native type form, and an additional type form originated from India (Beech strain).The mosquitoes were experimentally infected with *P. berghei* to compare their susceptibility to parasitism. Anti-mosquito midgut antiserum was then raised in BALB/cs mice immunized against gut antigens from the most susceptible form of *A. stephensi* (Beech strain), and the efficacy of the antiserum was assessed in transmission-blocking assays conducted on the least susceptible mosquito biological form.

**Results:**

The susceptibility of different biological forms of *A. stephensi* mosquito to *P. berghei* was specifically inter-type varied. The Beech strain and the intermediate form were both highly susceptible to infection, with higher oocyst and sporozoite infection rates than intermediate and mysorensis forms. The oocyst infection, and particularly sporozite infection, was lowest in the mysorensis strain. Antiserum raised against midgut proteins of the Indian Beech type form blocked infection in this mosquito population, but it was ineffective at blocking both oocyst and sporozoite development in the permissive but geographically distant intermediate form mosquitoes. This suggests that a strong degree of incompatibility exists between the mosquito strains in terms of midgut protein(s) acting as putative ookinete receptors.

**Conclusions:**

The incompatibility in the midgut protein profiles between two biological forms of *A. stephensi* demonstrates a well-differentiated population structure according to geographical origin. Therefore, the design of potential transmission-blocking strategies should incorporate a more thorough understanding of intra-species variations in host-parasite interactions.

## Introduction

Many different strains and species of malaria parasite exist, and this is also true for their mosquito hosts. Therefore, various levels of host-parasite compatibility can occur, and the extent of this compatibility determines the success of infection transmission. The vectorial capacity of malaria vectors for different *Plasmodium* species is greatly influenced by the diverse characteristics of the plasmodial parasite and eco-ethological attributes of the mosquito [1]. Such variations in vectorial capacity between individuals and strains within vector populations have been reported in *Anopheles gambiae* Giles, 1902 [2], *A. maculipennis* [3], *A. albimanus* [4], [5] and *A. culicifacies* [6]. Few mosquito vectors exhibit total refractoriness or susceptibility but rather (depending on geographical origin of both the parasite and the mosquito) intermediate susceptibility [5], [7]–[9]. For instance, *P. falciparum* parasites can be transmitted more successfully through a local, indigenous, mosquito species rather than a non-local species [9]. Understanding evolution in host–parasite interactions in spatially structured populations is important in both basic and applied biology, and it may impact significantly on the successful development and deployment of malaria transmission-blocking vaccines (TBVs), particularly if the goal is a global TBV that works across all anopheline species [10]. Furthermore, variation in interactions between parasites and their hosts is thought to be a major force in the co-evolutionary process [11] and in generating biological diversity [12].

*Plasmodium* is particularly vulnerable to population losses at three major stages during its development in the mosquito. The developmental transitions from gametes to ookinetes in the midgut lumen, oocyst development in the midgut epithelium, and sporozoite migration to the salivary glands via the haemocoel, are all at risk [13]–[15]. The relative severity of these losses varies between different parasite–mosquito species combinations, so that different mosquito species may show different permissiveness to a certain *Plasmodium* species and *vice versa* [16]. Different wild-caught members of the species complex *A. culicifacies*, for example, exhibit mean oocyst numbers ranging from 25 to just 2 depending on the mosquito species, and sporozoite infection rates ranging from 56% to less than 1%, after being fed with *Plasmodium vivax* [17]. Determinants of mosquito/malaria specificity still remain poorly understood, yet they are of significance to the success of TBVs.

Due to the focal nature of malaria transmission, TBVs have the greatest potential to eliminate malaria on a regional scale. They may also contribute to malaria elimination in larger areas if used in combination with other interventions, and even to prevent a looming epidemic [18]. TBV-induced immunity blocks the fertilization or the subsequent development of malaria parasites in the mosquito midgut, using antibodies generated in vaccinated human hosts. Nevertheless, such strategies could be more effective if host/parasite inter-species compatibilities are well understood.

However, the sporogonic development of malaria parasites has been studied extensively and many factors such as physical, biochemical and genetic factors that influence the parasite's development in the mosquito have been described. A secreted midgut peritrophic matrix can provide a relatively effective barrier limiting ookinete invasion of the midgut epithelium, however the extent to which this is successful depends on the mosquito species [19], [20]. A fuller understanding of the biochemical basis of subsequent ookinete/mosquito midgut cell ligand interactions is still incomplete although it is known that negatively charged oligosaccharides such as sulphated chondroitin polysaccharides and glycosaminoglycans on gut proteins, occur in the mosquito *A. stephensi* [21], [22]. There is mounting evidence that different mosquito species exhibit different and specific mixtures of potential ookinete-binding ligand glycotypes, whose assembly is controlled by a complex series of enzymatic reactions in the Golgi apparatus [22]. Ookinete invasion may also be assisted by midgut-derived aminopeptidases such as AgAPN1 [21], and a plethora of new potential TBV targets has also been identified from glycosylphosphatidyl inositol-anchored proteins originating in the lipid domain of *A. gambiae* midgut brush-border microvilli [23]. More recently, the expression of a recombinant anopheline alanyl aminopeptidase N (rAnAPN1) antigen (in *Escherichia coli*), is under investigation as a promising candidate [24]. However, the extent to which the success of blocking such antigens may be species-specific, is currently unclear, and there is further evidence that ookinetes may evade transmission blocking by employing multiple ligand binding interactions [21].

In addition, the bacterial flora found in the mosquito midgut lumen may adversely affect the development of the parasite, and this putatively offers yet another level of specificity at the level of the individual mosquito. Recently, it was shown that midgut bacteria can inhibit *P. falciparum* oocyst formation in *A. gambiae* but the extent of inhibition was bacterial species–dependent and also the active replication of the bacteria was required for parasite inhibition [25].

*Anopheles stephensi* and *P. berghei* are both amenable to molecular and biochemical studies [26]–[28], making this a powerful model system for understanding aspects of mosquito-plasmodium interaction [29]. *A. stephensi* is incriminated as a major vector of malaria in the Indo-Pakistan sub-continent as well as Iran [30]. In addition, this species exhibits a strong preference for human blood in south and southeastern Iran [31], [32]. Based on morphological characteristics of the egg (length, breadth, number of ridges on the egg float), three biological forms have been reported in this mosquito species: *A. stephensi* type form, intermediate form, and *A. stephensi* mysorensis [33]. However, crossing experiments between geographical strains [34]–[36] or biological forms of *A. stephensi* [32] have shown that these mate readily in the laboratory and produce viable offspring, with no evidence of post-copulatory barriers or male sterility in the F1 generation. Suleman *et al.* [37] demonstrated intra-specific variation in the reproductive capacity of *A. stephensi* but there was no evidence that this species constituted a species complex. The mysorensis form of this species has been recognized as the main vector of malaria in southeastern Iran [38]–[39], while in India it is considered to be a rural species with poor vectorial capacity for both vivax and falciparum malaria. In contrast, the type form is recognized as an efficient vector of urban malaria in India [32]. Even though *A. stephensi* is recognized as a competent laboratory host for the murine parasite *P. berghei*, there is no published comparison of the competency of different biological forms of this mosquito with *P. berghei*. In the present study, therefore, the susceptibility of three biological forms of *A. stephensi* to *P. berghei* was surveyed. Anti-midgut antiserum was raised against midgut antigens of a susceptible strain (Indian Beech type form) and its specificity/cross-reactivity with a geographically distant but approximately equally permissive form of *A. stephensi* (Iranian intermediate form) was assessed in terms of its potential to block parasite invasion in the midgut and thus transmission-blocking activity.

## Results

### Susceptibility of *A. stephensi* biological forms to *P. berghei*

#### Infection rate

**Figure 1:**
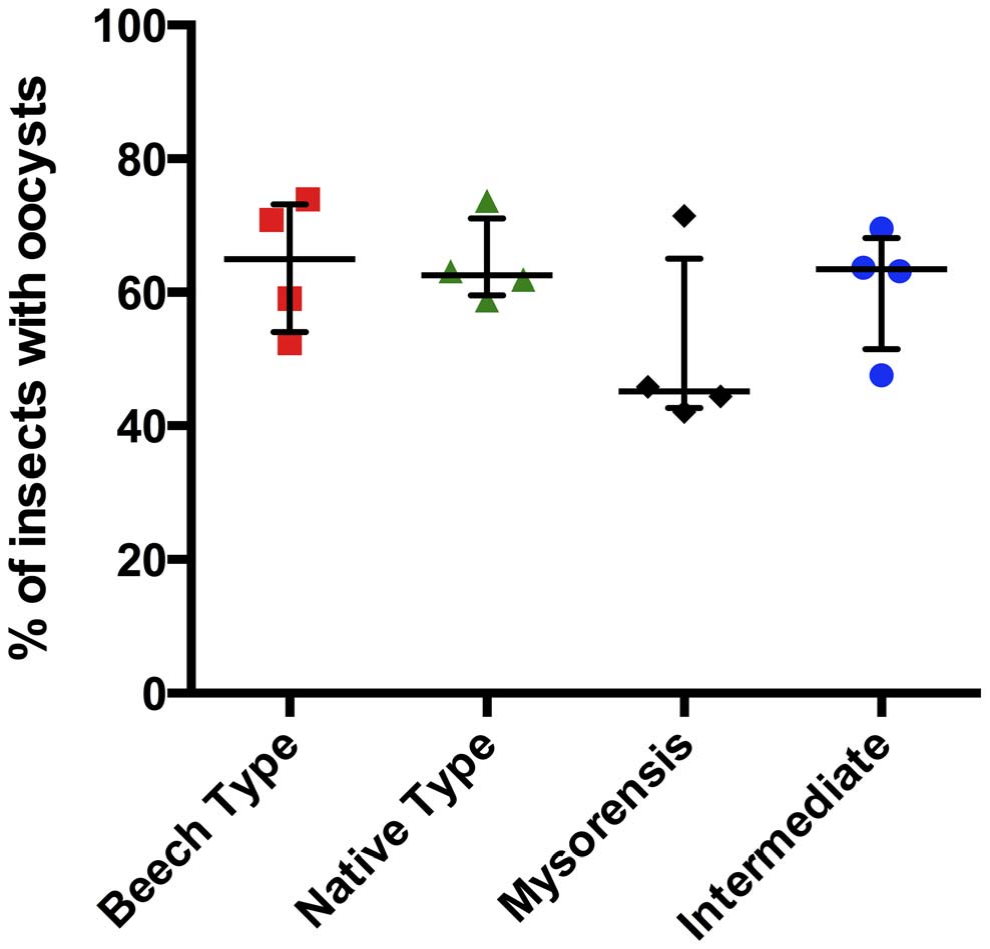
Prevalence of *Plasmodium berghei* oocysts in different biological forms of *Anopheles stephensi*. All batches of mosquitoes were artificially fed on BALB/c blood with equalized parasitaemas. The percentage of fed mosquitoes exhibiting oocyst formation in their midguts was then determined. Each bar represents the geometric mean of four experiments, +/− 95% confidence intervals (*n* = 4; each experiment included 17–24 fed mosquitoes per test group). There were no significant differences in oocyst prevalence between the forms of mosquito.

The four *A. stephensi* mosquito populations were fed a blood meal containing *P. berghei* and then an overall infection rate was calculated for each biological form as the percentage of mosquitoes with oocysts present in their midgut (irrespective of the intensity of infection). As indicated in Fig 1, minor strain-specific variations in the infection intensity were observed, but none of the differences was statistically significant (*p*>0.05; 1-way ANOVA with Kruskal-Wallis post-test). Both the type forms (Beech type and native Iranian type) were marginally more susceptible to P. berghei (respectively, 64.1% and 64.4% mean infection rates) than the intermediate and mysorensis forms (61.0% and 51.0%, respectively; Fig 1).

**Figure 2:**
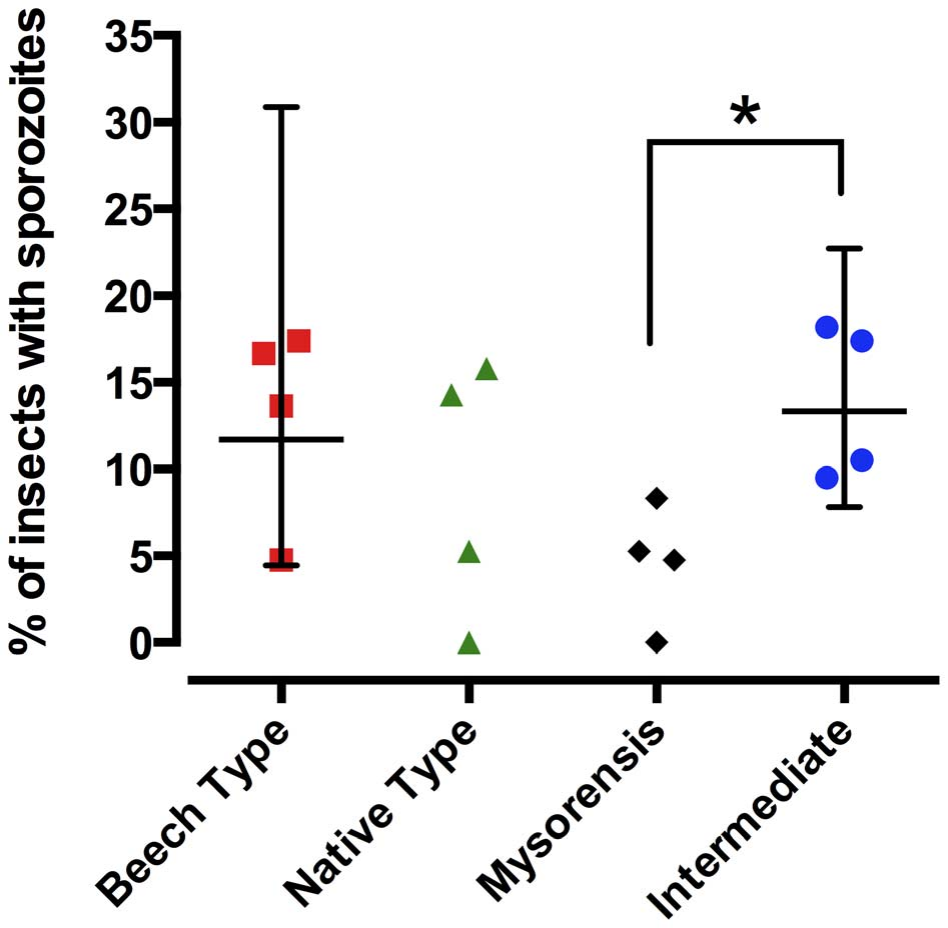
Prevalence of *Plasmodium berghei* sporozoites in different biological forms of *Anopheles stephensi*. All batches of mosquitoes were artificially fed on BALB/c blood with equalized parasitaemas. The percentage of fed mosquitoes with sporozoites formation in their salivary glands was then determined. Each bar represents the geometric mean of four experiments, +/−95% confidence intervals (*n* = 4; each experiment included 17–24 fed mosquitoes per test group). Asterisk denotes significance (P = 0.0286, non-parametric Mann-Whitney t-test).

The salivary gland infection rate was determined in the same way as above, but by identifying the presence of sporozoites (Fig 2). In all the mosquito populations, significantly fewer mosquitoes exhibited salivary gland infection than had acquired oocysts (all comparisons *p*<0.001; paired t-tests), signifying substantial parasite losses at the sporogonic developmental bottleneck irrespective of the mosquito form. The highest percentage of infected mosquitoes belonged to the intermediate and Beech type (13.9% and 13.1% of insects had sporozoites, respectively), while the mysorensis strain was the most refractory (4.6% of insects with sporozoites). In this case, the difference in infection rates between the intermediate and mysorensis mosquito forms was also significant (P = 0.0286, non-parametric Mann-Whitney t-test; Fig 2). The native type forms exhibited moderate susceptibility (8.8% infection rate).

#### Infection intensity

**Figure 3:**
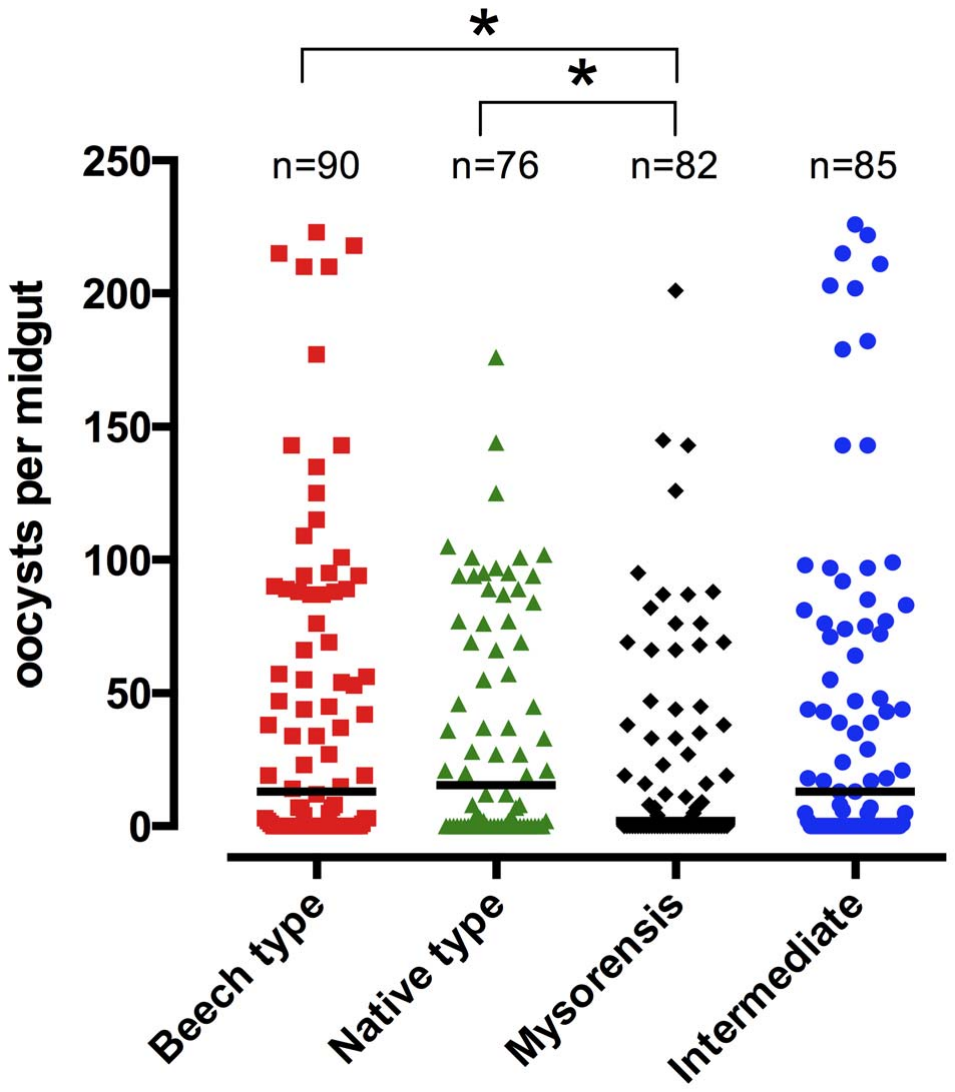
Intensity of *Plasmodium berghei* oocyst infection per midgut in different biological forms of *Anopheles stephensi*. All batches of mosquitoes were artificially fed on BALB/c blood with equalized parasitaemas. Each dot represents the number of oocysts in an individual midgut, and the graph shows pooled data from four experiments. The horizontal bars indicate median infection intensity. Asterisks denote significant differences at *p*<0.05 between the different mosquito forms (Mann-Whitney non-parametric t-test). The pooled data exhibit significantly different variances. Additional analyses were performed (by Kruskal-Wallis 1-way ANOVA with Dunn's multiple comparisons post test) on log-transformed oocyst counts from infected midguts (ignoring uninfected midguts), and did not find significant differences between the mosquito types.

The relative intensities of parasite infections also varied between the infected members of the different *A. stephensi* forms, with a weak positive correlation between the mean oocyst count per experiment and sporozoite prevalence for each mosquito type (R^2^_mysorensis_  = 0.232, R^2^_intermediates_ = 0.157, R^2^_Beech type_ = 0.19 and R^2^_native type_ = 0.003). The highest oocyst counts were observed in the Beech population, followed by the intermediate population (Fig 3). Very high oocyst loads (>120 per blood-fed midgut) were found in ca. 11.8% of the intermediate form and 11.1% of the Beech population, compared with ca. 4.9% of blood-fed mysorensis mosquitoes and ca. 3.9% of the native type form population. In contrast, ca. 35.6% of fed females in the Beech population, 38.8% in the intermediate, 35.5% in native type form and 48.8% in mysorensis were completely refractory to oocyst development in the midgut despite receiving an infected bloodmeal.

The mean numbers of oocysts per (blood fed) midgut in Beech, intermediate, native type form and mysorensis were 44.58, 44.86, 36.55 and 25.11 respectively, and the medians were 13.0, 13.0, 15.5 and 2.0 respectively (Fig 3; pooled data for 4 experiments). In considering both infected and uninfected mosquitoes combined, the oocyst intensities were significantly different between the mysorensis and Beech type forms (*p* = 0.028), and the mysorensis and native type forms of the mosquitoes (*p* = 0.043; Mann-Whitney t-test). However, the data exhibited significantly different variances (Bartlett's test and F value analyses). An analysis was therefore also performed for log-transformed oocyst counts per infected midgut (ignoring uninfected midguts) which did not show any significant differences between the different mosquito forms (*p*>0.05; Kruskal-Wallis 1-way ANOVA with Dunn's multiple comparisons post-test). Overall, however, the mysorensis form was judged to exhibit the greatest refractoriness to *P. berghei* in these experiments. Based on combined susceptibility to oocyst and sporozoite infections, the most susceptible mosquito population, capable of transmitting the most parasites to a new host, was the intermediate form.

### Transmission-blocking assay

**Figure 4:**
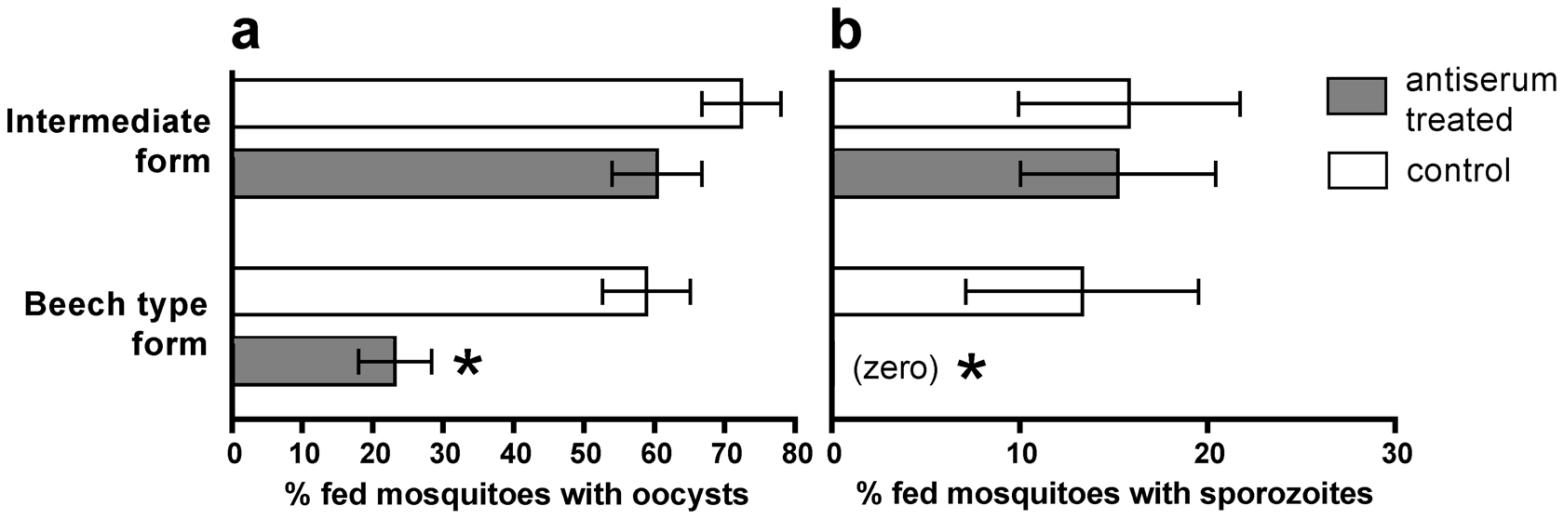
Effects of anti-mosquito midgut antiserum on the prevalence of *P. berghei* oocysts and sporozoites in two populations of *A. stephensi*. Antiserum was raised against midgut antigens from the Beech type form *of A. stephensi* and then a mixture of antiserum and *P. berghei* were fed to both the Beech type form and to intermediate form mosquitoes. The bars represent the percentage of fed mosquitoes exhibiting (a) oocysts in their midguts, and (b) sporozoites in their salivary glands. The mean of three replicate experiments is presented, +/− SEM. Asterisks denote significance at *p*<0.05 (calculated by the Mann-Whitney non-parametric t-test).

An antiserum was raised against midgut antigens from the Beech type form mosquitoes, which in this study were judged to be very permissive to *P. berghei*. This antiserum recognized the midgut proteins of Beech population as it significantly (*p*<0.05) decreased the prevalence of oocyst infection in this mosquito population compared with the antiserum-free controls (Fig 4a). Furthermore, sporozoite infections were completely interrupted in the Beech population following antiserum treatment (Fig 4b). In strong contrast, the administration of antiserum did not affect the prevalence of oocyst infection in the intermediate form of *A. stephensi* compared with its control, with 15.2% of the dissected mosquitoes from the test group still containing sporozoites in their salivary glands and 15.8% of the controls (Fig 4b).

## Discussion

In this study, subtle variations were observed in the *P. berghei* infection rate within different biological forms of *A. stephensi*, and these variations were most noticeable at the sporozoite stage of infection. Variations in oocyst infection rates, the mean number of infected midguts and salivary glands, and the significant differences in oocyst intensity data variances, all indicate a differentiated population structure according to geographical origin [40]. Although the observed differences in permissiveness to *P. berghei* were relatively minor between the mosquito populations, more striking was the finding that antiserum raised against midgut antigens from one mosquito population was unable to recognize midgut antigens from a geographically distant population, even though both those mosquito strains exhibited almost equal permissiveness to the parasite. Whether this differentiation is solely due to geographical separation, or indicative of a biological barrier, remains an open question.

Although there were not great differences between the mosquito populations in terms of parasite permissiveness, nevertheless, antiserum raised against midgut antigens from one mosquito population was unable to recognize midgut antigens from a geographically distant population, even if both mosquito strains exhibited almost equal permissiveness to the parasite.

It should, however, be pointed out that the model parasite *P. berghei* is not encountered naturally by *A. stephensi* so the comparative susceptibility of these four populations to natural parasites has not yet been studied. Among the four mosquito populations, the Beech strain (Indian type form) and intermediate forms of *A. stephensi* exhibited high infection rates as well as oocyst and sporozoite loads, while the mysorensis form was found to be the least susceptible. The mysorensis form is a major vector of vivax malaria in Iran but different parasite species may trigger different responses in the mosquito as *P. vivax* versus *P. burghei* may elicit different responses in the same mosquito vector.

This indicates that the mysorensis form might possess a better-developed mechanism of tolerance to *P. berghei* sporogonic development, lacking in the other biological forms studied, which exhibit enhanced formation of ookinetes and more efficient traversal of the midgut. As previously reported in interactions between some mosquito vectors of *Plasmodium*, co-evolution may lead to an increase in parasite adaptation against the insect immune system [41], [42]. More recently, local adaptation within vector populations and their *Plasmodium* populations has been reported [40] by comparing the level of infection within both sympatric and allopatric *P. falciparum*/*A. gambiae* combinations in each of two separate geographical areas in Burkina Faso and Cameroon. There is contradictory evidence that both allopatric and sympatric infected mosquitoes may be more refractory, but the most detailed studies (Harris et al) using wild or recently-captured mosquito populations do tend to suggest that sympatric mosquitoes are more resistant to local Plasmodium strains[40].

However, although we have focused on effect of mosquito geographical population on parasite development, it should also be noted that ookinete development may be affected by physical and biochemical barriers [41]–[44] as well as the innate immunity of the mosquito vector [45].

In the current study, a positive correlation was observed between the prevalence of infection in the mosquitoes and the intensity of oocyst formation. It has previously been proposed that the impact of transmission-blocking substances manifests mainly in a reduction in infection intensity, but not on prevalence under conditions of high oocyst intensities, whereas a rapid reduction in prevalence occurs under conditions of low oocyst intensities [46]. There is a relationship between mean oocyst count and mean salivary gland sporozoite numbers, as it has been shown that at low oocyst count prevalence (less than 50%) the sporozoite infection significantly cut in *A. stephensi* where the oocysts counts were much higher (about 90%), even substantial reductions in oocyst numbers may be insufficient to prevent sporozite development and hence prevalence is largely unaffected [40].

In the present study, we also found a positive correlation between increased ookinete development and greater salivary gland infection by sporozoites. The oocyst intensity analyses also suggest that initial factors affecting success or failure of parasite survival in the midgut after ingestion are type-specific and varied than later factors that determine how many oocysts eventually form in the midgut. These observations are interesting and provide an insight into parasite-vector transmission dynamics, by suggesting that the initial physical and biochemical barriers in the mosquito midgut are a greater hurdle for development of the parasite than are factors in the haemocoel compartment.

To address the hypothesis that the midgut presents the more formidable barrier to sporogonic development, we tested the compatibility of parasite proteins for the midgut antigens of two similarly permissive mosquito populations from geographically distant habitats (the intermediate form as a native population, and the Beech type as more adapted vector to the parasite). Importantly, no considerable antigenic cross-reactivity was observed between midgut proteins of type form (Beech population) and intermediate form mosquitoes. The antiserum raised against proteins extracted from the Beech type form midgut, while clearly able to block sporogonic development in the Beech mosquitoes, did not significantly inhibit oocyst development in the midguts of intermediate form mosquitoes and had no impact on sporozoite invasion of their salivary glands. We therefore suspect an incompatibility in the midgut protein profiles between these two groups of mosquitoes, which may act as determinants of infectivity and be exploited by *P. berghei* when infecting the type form. It has been shown previously that high titres of antisera raised against subcellular fractions of *A. stephensi* midgut microvillar preparations, inhibited survival, fecundity and even *P. berghei* transmission [47], [48]. Our data provided similar results with respect to the Beech (donor) population but highlight that the transmission blocking effect was only successful in the mosquito population from which the antigens were donated,

Antibodies targeting the mosquito midgut have long been considered an important possible strategy for the development of mosquito-based TBVs, and much research is directed towards this goal [10], [49], [50]. Compared with other potential malaria vaccines, there is less risk of TBV-resistant mutant parasites emerging because TBVs target the parasite's sexual stages that do not multiply, and that are present in very low numbers [10]. We show here, however, that antigenic variation in mosquito strains or biological forms may significantly impact the development of such mosquito vaccines, and so understanding the mosquito/parasite compatibilities could have important implications for the design of potential transmission-blocking strategies. While TBVs may be efficacious on a regional scale, further studies of the intra-variation of any mosquito species infected with different parasites are required to determine directly if, and how, a universal transmission blocking vaccine should be designed. In addition, a better understanding of the limitations imposed by mosquito susceptibility would be advantageous in predicting the potential spread of these undesirable phenotypes and allow cost-effective vector control measures to be implemented.

In conclusions, this study highlights antigenic variation in mosquito biological forms that could significantly impact the development malaria TBVs. The design of potential transmission-blocking strategies should incorporate a thorough understanding of intra-species variations in host-parasite interactions.

## Methods

### Mosquitoes

Four colonies of mosquito consisting of three different biological forms of *A. stephensi* were reared under uniform conditions for larval and adult nutrition, temperature (28±2°C), humidity (70±10%) and photoperiod (12 h light-dark cycle). These three Iranian mosquito populations were originally collected from three distinguishable, different, ecological zones and colonized in an insectary before this *study.* The native Iranian type form (Kazeron population) was collected from the Kazeron area (Fars province) located in the northern region of the Zagros mountain chain (51° 39′ N and 29° 37′ E), while the intermediate form (Bandar-Abbas population) originally came from Bandar-Abbas (59° 15′ N and 25° 24′ E) situated at the south slope of the Zagros mountains, north of the Persian Gulf. The mysorensis population (Iranshahr population) was originally collected from southeastern Iran, Baluchistan province, Iranshahr (41° 60′ N and 12° 27′ E) near the Pakistan border. The distance between Kazeron (north of Zagros) and Bandar-Abbas (south of Zagros) is more than 550 km, and the distance between Iranshahr (in the east of Iran) and Bandar-Abbas is about 700 km. The Beech strain was originally collected from Pakistan (an additional type form i.e. SDA500 strain originating from Pakistan, known as the Beech strain which was provided in 2005 by Professor P.F. Billingsley, Sanaria, Inc.).

Larvae were grown in bowls at a density of 300 larvae per 500 ml of distilled water with 0.01% table salt, and fed on fish food. The pupae were transferred to cages made of muslin cloth before eclosion to the adult stage. Adults were fed on 10% fructose and the females were artificially blood-fed on defibrinated cow blood. For experiments, separated colonies of mosquitoes were starved for 12–18 hours before feeding artificially on a blood meal containing *P. berghei*.

### Parasite preparation

*P*. *berghei* ANKA 2.34 strain (also donated by P.F. Billingsley) was maintained by cyclical passage through BALB/c mice and *A. stephensi* Beech strain. To maximize exflagellation, BALB/c mice were treated with 100 µL 1% phenylhydrazine 3 days prior to injection of *P. berghei*. Subsequently, 300 µL of infected blood was injected i.p. in each BALB/c mouse. Three days post infection, murine parasitemias were verified and exflagellation surveyed by mixing a drop of infected blood with ookinete culture medium (RPMI 1640 with L-glutamine and 25 mM HEPES, 2 g/L sodium bicarbonate, 50 mg/L hypoxanthine, 50000 U/L penicillin and 50 mg/L streptomycin; pH 8.3, filter sterilized and supplemented with 10% FCS). Mice with an exflagellation rate of more than 20/field under ×40 microscopic magnification were used for feeding the mosquitoes. All infected bloods were mixed to equalize the parasite concentration fed to each mosquito batch.

### Susceptibility of biological forms of *A. stephensi* to *P. berghei*

Non blood-fed adult female mosquitoes 4–7 days post-eclosion were collected from stock cages of each biological form and placed into mesh-topped containers 1 day prior to the feed. Four replicate batches, each comprising at least 25 females from each mosquito colony, were provided with only distilled water for 24 hrs prior to blood feeding. All mosquitoes were fed via an artificial membrane feeder with a circulating water bath maintained at 37°C, using mixed blood from four gametocytaemic mice. Mosquitoes were allowed to feed for 30 minutes in an environment with an air temperature of 19–21°C. A sample of the infective feed was examined by light microscopy to confirm exflagellation. Unfed mosquitoes were discarded and the fully engorged females were carefully maintained at 19–21°C and 60%±10 humidity to promote gametogenesis, and fed on 10% fructose until the mosquitoes were dissected. Midgut dissection was performed on day 10 post-feeding, and the midguts were stained with mercurochrome then examined by light microscopy for the presence of oocysts. Infection rates of mosquitoes (*prevalence*), mean number of oocysts in the midgut (*intensity*) as well as infection rate of the mosquitoes' salivary glands were calculated. Finally correlation between the mean oocyst count per experiment and sporozoite prevalence for each mosquito type was calculated using GraphPad Prism (v. 4 & 6, GraphPad Software, La Jolla California, USA; www.graphpad.com).

### Antigen/antiserum preparation

Midguts antigens from the susceptible type form (Beech) mosquitoes were prepared for antiserum production. Midguts of 3686 female were dissected in PBS and homogenized at 4°C, then centrifuged at 16000 g at 4°C for 20 minutes. The pellet was resuspended in 1% Triton X-100 plus 1% Tween®20 in PBS (0.01 M phosphate buffered saline, pH 7.4) and centrifuged again at 6000 g at 4°C for 20 minutes. Then the sample was deglycosylated as described before (32). Briefly, the pellet was re-suspended in sodium acetate and centrifuged, the pellet treated with 10 mM periodic acid in 50 mM sodium acetate for 1 hour at room temperature (RT) in the dark and centrifuged. The pellet was suspended in 1% glycine in Tris-HCl (0.5 M) in saline (0.15 M) and after 30 mins in RT, centrifugation was repeated and the pellet suspended in Tris-HCl-saline. Protein concentration was adjusted to 400 µg/ml. The final produced was mixed with Alum Adjuvant at 1∶1.

Twenty BALB/c mice (Razi Institute, Karaj, Iran) were divided into two equal groups; every mouse in the test group was injected 4 times subcutaneously with 100 µL of the preparation (containing 20 µg of protein) at three-week intervals, while all mice in the control group received PBS.

The sera of BALB/c mice were collected 10 days after final antigen injection and antiserum titers were tested using ELISA as detailed below.

### ELISA-based assessment of antiserum titer

Deglycosylated midgut preparation was diluted with coating buffer to 2 µg/ml of protein and loaded into the wells of ELISA polystyrene 96 well flat bottom microplates (Nunc-Immuno Plate Maxisorp, Denmark) in 50 µL volumes, incubated at 4°C overnight and washed 3 times with 0.05% PBS- Tween®20 (PBS-T) and stored at −20°C. When in use, 200 µL of 1.5% bovine serum albumin was added to each well and incubated for 30 mins at RT, washed, and 50 µL of 1∶10, 1∶100 and 1∶1000 diluted sera from test and control group mice were added and incubated for 1 hour at RT. Wells were washed again and 50 µL of 1∶20000 diluted anti-mouse-IgG conjugated peroxidase (Razi Biotech Co.) was added and kept for 1 hour at RT. The wells were washed and 50 µL of substrate (3,3′,5,5′–tetramethylbenzidine, Razi Biotech Co.) was added to each well. After 30 mins at RT, 25 µL of 5% sulfuric acid was added and a positive color-change reaction was gauged by visual inspection. Sera from the test group mice exhibited strongly positive reactions towards the midgut antigens, even at the highest tested dilution (1∶1000). Sera from the control group were negative.

### Transmission-blocking assay

Experiments described earlier in this study demonstrated that the type form (Beech) mosquito population is relatively susceptible to *P. berghei* infection but also has an approximately equal permissiveness to the geographically-distant intermediate form. A transmission-blocking assay was therefore conducted to assess the efficacy and specificity of antiserum raised against Beech form midgut antigens, by also testing the antiserum on the intermediate form.

*P. berghei* infected blood was taken by cardiac puncture from infected mice using heparin as the anticoagulant. Following confirmation of more than 20 exflagellations/high power field of microscopic observation, the blood was centrifuged, the plasma and buffy coat layers were discarded, and the packed red blood cell layers of all mice were pooled. Separately, equal volumes of infected red blood cells and pooled serum from test and control mice were mixed to make blood samples with or without anti-midgut antiserum. Non-fed female mosquitoes from Beech type and Intermediate forms were divided into two containers each containing 80–100 mosquitoes, and were fed from blood samples with antiserum (test mosquito group) or without antiserum (control mosquito group) whilst maintaining the temperature of the samples at 37°C and then immediately after feeding, non- or partially fed mosquitoes were discarded, and fully engorged individuals were transferred to a 20°C incubator with 12/12 hr periods of dark and light, and fed with 10% fructose *ad libitum*. Ten days after feeding, midguts of approximately half the mosquitoes in each container were dissected to evaluate the prevalence and intensity of oocysts as described above. Salivary glands of the remaining mosquitoes were dissected 20 days post-feeding to assess the presence of sporozoites. This experiment was performed three times.

### Statistics

Non-parametric tests were used to compare the prevalence of oocyst and sporozoite infection in different groups (1-way ANOVA Kruskal-Wallis with Dunn's multiple comparisons post test). For the intensity of oocyst infections, the data were abnormally distributed and furthermore the variances were significantly different (F value test), meaning that neither Kruskal-Wallace 1-way ANOVA nor Mann-Whitney t-tests were entirely appropriate. Therefore, although the Mann-Whitney t-test was performed on pooled oocyst counts per midgut from infected and uninfected mosquitoes (as used by [23]), we also log-transformed oocyst counts per infected midgut (ignoring uninfected samples) to equalize variances, and then performed ANOVA as described above. Paired t-tests were used to detect significant differences between oocyst and sporozoite prevalences within the same mosquito population. Transmission-blocking assay data for oocyst and sporozoite prevalences were compared using the Mann-Whitney non-parametric t-test; differences between antiserum-fed versus control insects were considered significant when *p*<0.05. Analyses were conducted using GraphPad Prism (v. 4 & 6, GraphPad Software, La Jolla California, USA; www.graphpad.com).

### Ethics statement

On behalf of, and having obtained permission from all the authors, I declare that(d)all relevant ethical safeguards have been met in relation to animal experimentation. Ethical approval was obtained from Ethics Committee of Tehran University of Medical Sciences. During this study, the researchers considered for animal welfare, including pain management, nutrition, laboratory animal diseases, justification for the number of mice in each cage as well as paying enough attention to correct laboratory procedures, anesthesia, and euthanasia.
